# Quality Characteristics of Meat Analogs through the Incorporation of Textured Vegetable Protein: A Systematic Review

**DOI:** 10.3390/foods11091242

**Published:** 2022-04-26

**Authors:** Allah Bakhsh, Eun-Yeong Lee, Chris Major Ncho, Chan-Jin Kim, Yu-Min Son, Young-Hwa Hwang, Seon-Tea Joo

**Affiliations:** 1 Division of Applied Life Science (BK21 Four), Gyeongsang National University, Jinju 52852, Korea; drbakhsh01@gmail.com (A.B.); ley9604@gmail.com (E.-Y.L.); ckswls090@gmail.com (C.-J.K.); dbals910@gmail.com (Y.-M.S.); 2 Department of Animal Science, Gyeongsang National University, Jinju 52852, Korea; chrismajor159753@gmail.com; 3 Institute of Agriculture & Life Science, Gyeongsang National University, Jinju 52852, Korea; philoria@hanmail.net

**Keywords:** meat analogs, textured vegetable protein, quality characteristics, systematic review

## Abstract

Meat analogs produced through extruded products, such as texture vegetable protein (TVP) with the addition of various plant-based ingredients are considered the products that have great potential for replacing real meat. This systematic review was conducted to summarize the evidence of the incorporation of TVP on the quality characteristics of meat analogs. Extensive literature exploration was conducted up to March 2022 for retrieving studies on the current topic in both PubMed and Scopus databases. A total of 28 articles published from 2001 to 2022 were included in the data set based on specific inclusion criteria. It appears that soy protein is by far the most used extender in meat analogs due to its low cost, availability, and several beneficial health aspects. In addition, the studies included in this review were mainly conducted in countries, such as Korea, the USA, and China. Regarding quality characteristics, textural parameters were the most assessed in the studies followed by physicochemical properties, and sensory and taste attributes. Other aspects, such as the development of TVP, the difference in quality characteristics of texturized proteins, and the usage of binding agents in various meat analogs formulations are also highlighted in detail.

## 1. Introduction

Meat analogs are quality products that resemble meat in appearance, taste, texture and up to an extent, nutritional values; they can be meat-free or partially replaced with a minor amount of meat [1]. Meat analog can also be referred to as imitation meat, faux meat, meat substitute, or mock meat [2]. Steady growth has been seen in demand for plant-based meat analogs in recent years due to a change in consumer behavior that is largely attributed to consumers’ consciousness toward healthy choices in food selection [3] as well ethical and sustainability factors related to meat consumption [4]. According to nutritional values, meat plays a vital role in human nutrition, and red meat consists of highly valued biological proteins with vitamins, iron, zinc, and other micronutrients [5]. However, it has been evident from previous literature that consumption of red or processed meat for a prolonged period causes type 2 diabetes, cardiovascular complications, and some forms of cancers [6,7]. Additionally, the major issues linked with the production of meat include excessive use of land and water resources, high risk of animal diseases, negative impact on terrestrial and aquatic biodiversity, emission of greenhouse gases, and other environmental hazards [8,9,10]. Additionally, the global production and consumption of red meat have increased manifolds due to rapid economic development and a surging population [11]. Current statistics reveal that by 2018 approximately 320 tons of meat were consumed worldwide and market expansion is predicted to be 15% in 2027 [12]. For this reason, policymakers are expecting a shift from unhealthy and health-hazardous ingredients towards more sustainable products, i.e., meat analogs.

Moreover, the consumption of soy-based meat alternatives has shown several advantages over red meat consumption, such as the reduction in obesity, low blood pressure, and cholesterol levels, and positive psychological effects on human health, as well meatless plant-based meat analogs address the issues regarding animal welfare [13,14,15]. Currently, the academic research focuses on two major types of analogs, cell-based meat [16,17] and plant-based meat replacements [2,18,19,20,21]. Moreover, third-generation meat analogs are formed from TVP which is a dry bulk commodity derived from soy protein [18,19,20,22]. The production of TVP is regulated through a special process of protein extraction from various plants sources with the appropriate structuring and extrusion processes [18,23,24]. The production of TVP is occur through the high/low moisture extrusion process, and the resultant product mimics the texture and taste of real meat up to some extent [24]. The rehydration process is needed for TVP to obtain a fibrous and spongy nature before consumption in different forms, such as nuggets, patties, or sausage analogs [25]. The reason for the current study focusing on soy-based TVP as a prime ingredient in meat analogs is due to its natural properties, e.g., cholesterol-free, low in fat, and low in calories [7].

A lenient flavor and taste of the meat analog product is a crucial factor for consumer acceptance [26]. For the flavoring of meat analogs, savory spicing, meat, and savory aromas, as well as their precursors, are currently used along with iron complexes (e.g., ferrous chlorophyllin or heme-containing proteins. A range of agents, such as reducing sugars, amino acids, thiamine, and nucleotides have been used to mimic these aromas in meat analog products producing chicken-like aromas and beef-like aromas from the same soybean-based Enzyme-Hydrolyzed Vegetable Protein by affecting the pH of the reaction. However, extrusion cooking practice is a multifaceted and complex operation. During the process of extrusion, composite reactions occurred and the elements added lost their aromatic and volatile components partially. Furthermore, flavor perception changes with product temperature, product refinement, and storage conditions. Moreover, the lack of animal meat flavor that consumers are familiar with and expect is another major hurdle to the progress of alternative products [27]. During the extrusion process, the beany odor is considered to be associated with lipid oxidation products, such as hexanal and methanethiol, and the bitter-astringent tastes, caused by isoflavones and saponins, could be a limitation to the effectiveness of soy protein as the basic ingredients for meat alternatives [28].

Another existing challenge for meat analogs is the recreation of the unique texture, organoleptic properties, and juiciness as similar to the traditional meat products. In contrast, the focus has been on the selection of plant protein to recreate the physiochemical properties of animal protein. Factors include the ability to encapsulate fat, their oil, water-holding capacity, gelling, and emulsifying properties, which can be evaluated through texture analysis. Instead of using various plant protein sources, different kinds of food additives also can enhance the textural properties of meat analogs. Hydrocolloids have gelling, thickening, emulsifying, and stabilizing properties due to their ability to interact with water, proteins, starch, and other components in food products. Generally, carrageenan, an algae-derived polysaccharide, xanthan gum, methylcellulose, and konjac mannan are been considered common types of hydrocolloids that are present in the meat analogs [29]. However, so far, the contents and results of studies on the quality characteristics of analogs related to TVP are complex and different, so it remains difficult to draw general conclusions. Therefore, the main objective of this systematic review was to add new evidence to the literature by summing up the current knowledge on the incorporation of TVP on the quality characteristics of meat analogs.

## 2. Materials Methods

The current systematic review was strictly conducted following the PRISMA (Preferred Reporting Items for Systematic Reviews and Meta-Analyses) guidelines [30].

### 2.1. Data Sources and Searches

An extensive literature exploration of studies evaluating the quality characteristic of meat analogs after partial or complete replacement with texture vegetable protein was conducted in PubMed and Scopus. The literature was explored in at least two different scientific repositories that widen the inclusion criteria of articles found in both databases [31,32]. The following query was used to retrieve potential studies to be included in the review: (texture vegetable protein OR TVP) AND (meat) AND (alternat* OR substitut* OR analog* OR fake OR mock OR faux OR imitat*). The literature search was focused on collecting studies that were published up to March 2022.

### 2.2. Study Selection

**Figure 1:**
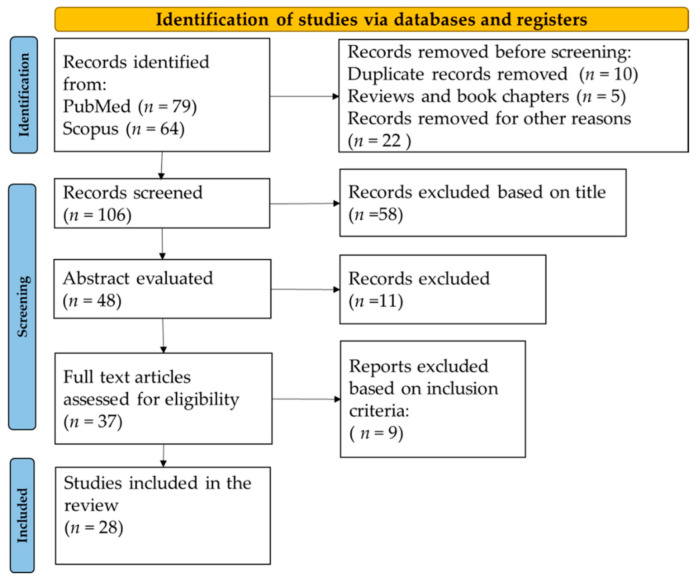
Flow diagram of the literature search process.

Two independent investigators conducted the study research while keeping in mind pre-specified inclusion criteria. The main criteria for study selection were: (i) articles should be published in peer-reviewed journals written in the English language; (ii) studies should evaluate at least one quality characteristic (texture, physicochemical properties, sensory properties, taste attributes); (iii) clinical studies evaluating the effects of textured vegetable protein on human health were excluded; (iv) studies should not be a survey conducted to evaluate consumer acceptance; (v) studies should focus on textured vegetable protein quality rather than any other parameters; (vi) studies that developed new technologies to assess textured vegetable protein quality were excluded. The study selection process is detailed in Figure 1.

### 2.3. Data Extraction

Data related to the studies included in the systematic review were collected by one investigator, then a second investigator checked carefully these data for accuracy. The following characteristic related to the studies were extracted: authors and year of publication; location of the included study; the type of textured vegetable protein used; quality characteristic evaluated; whether or not the TVP was incorporated totally or partially; when partial incorporation, what type of meat was included in the formulation; the binding agent used or not and finally the type of final product manufactured. When any disagreements happened during the data collection, a third investigator was consulted to resolve the issue.

### 2.4. Data Synthesis and Analysis

A meta-analysis was not conducted in the current review because no studies conducted had the same formulation. Thus, the authors did not find it suitable to pool results from studies with this level of dissimilarity. Indeed, there was a plethora of binding agents as well as different types of components included in the formulations of the meat analogs (gelatin, starch, molasses, mushrooms, etc.). Therefore, as recommended by the Cochrane handbook for systematic reviews of interventions only a qualitative synthesis of the data extracted from the studies was conducted [33]. The “*webr*” package of the R software (v.4.0.3) (R Core Team, 2020, R Foundation for Statistical Computing, Vienna, Austria) was used to draw the pie donut charts based on the data collected from studies. Bar plots and world maps were drawn using Tableau Desktop 2021 (Tableau software, 2003, Salesforce, Seattle, WA, USA).

## 3. Results

### 3.1. Study Selection Workflow

A total of 143 records were identified after consulting PubMed and Scopus databases. After removing 10 duplicates, excluding five reviews, and excluding 22 other articles consisting of book chapters or conference abstracts without full text, 106 articles remained. Thereafter, title and abstract screening were performed and led to 37 articles eligible for full-text screening. After carefully reading full texts and excluding articles not meeting the inclusion criteria, 28 articles published from 2001 to 2022 were finally included in the systematic review.

### 3.2. Study Characteristics

Table 1 shows the detailed characteristics of the articles selected for the systematic review.

**Figure 2:**
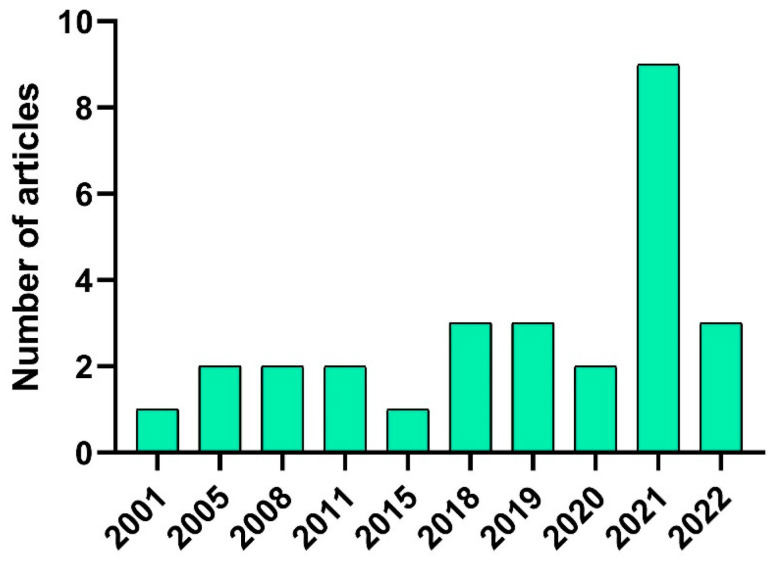
The number of articles published per year is included in the systematic review. The chart was constructed based on *n* = 28 articles.

It appears that the majority of studies focused on the use of soy protein only (*n* = 14). In addition, some other studies evaluated the combination of soy protein with other proteins, such as pea (*n* = 4), insect (*n* = 1), rice (*n* = 1), and wheat (*n* = 1). Concerning the chronological apparition of the studies, the oldest article included in the review was dated 2001 and the newest was released in 2022 (Figure 2).

**Figure 3:**
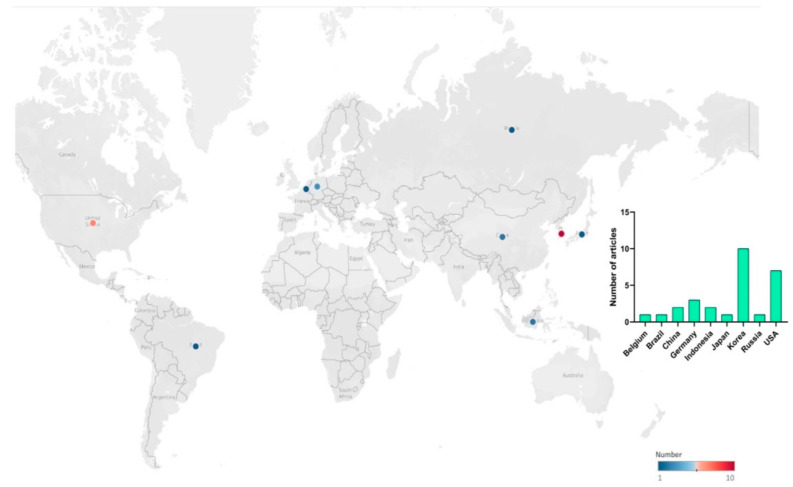
Map showing the number of articles included in the systematic review per country. The chart was constructed based on *n* = 28 articles.

Moreover, the highest number of articles were published in the years 2021 (*n* = 11) followed by 2018, 2019, and 2022 each year (*n* = 3), respectively. Geographically speaking, the preponderance of studies was conducted in Asia and North America (Figure 3). More precisely, the highest number of studies were conducted in Korea (*n* = 10) and the USA (*n* = 7).

**Figure 4:**
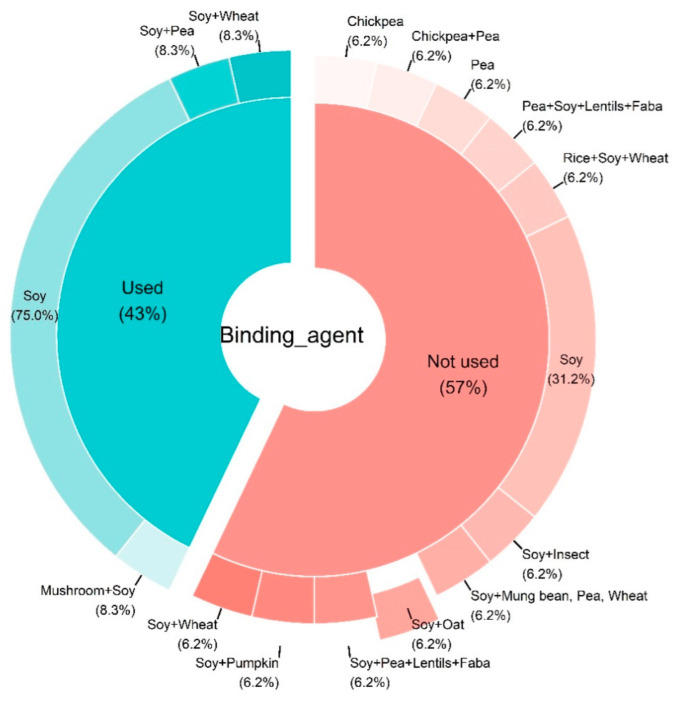
Pie donut chart summarizing the type of textured vegetable protein according to the used biding agent from the studies included in the systematic review. The chart was constructed based on *n* = 28 articles. Abbreviations: C.c: Coprinus comatus.

An in-depth analysis of the different study designs revealed that 43% of the studies used at least one binding agent in their meat analog formulation while 57% did not. Figure 4 shows that the majority of studies using binding agents were using only soy protein (75%).

**Figure 5:**
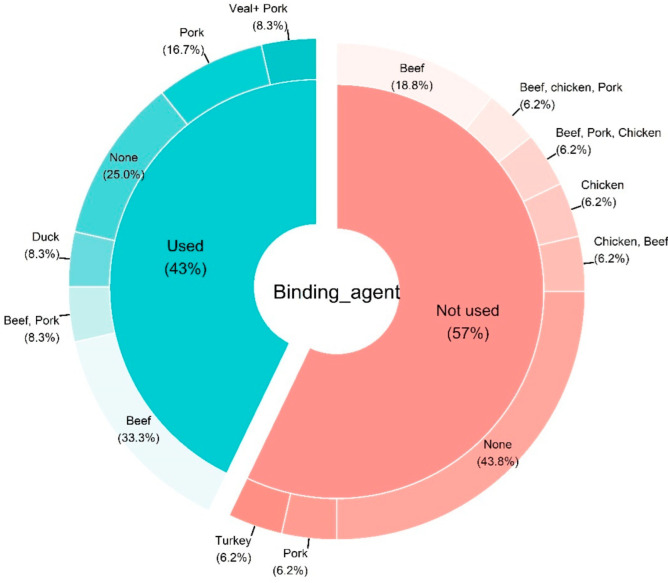
Pie donut chart summarizing the type of meat according to the used biding agent from the studies included in the systematic review. The chart was constructed based on *n* = 28 articles.

Moreover, beef was predominantly used whether authors included (33.3%) a binding agent or not (18.8%) in their formulations (Figure 5).

**Figure 6:**
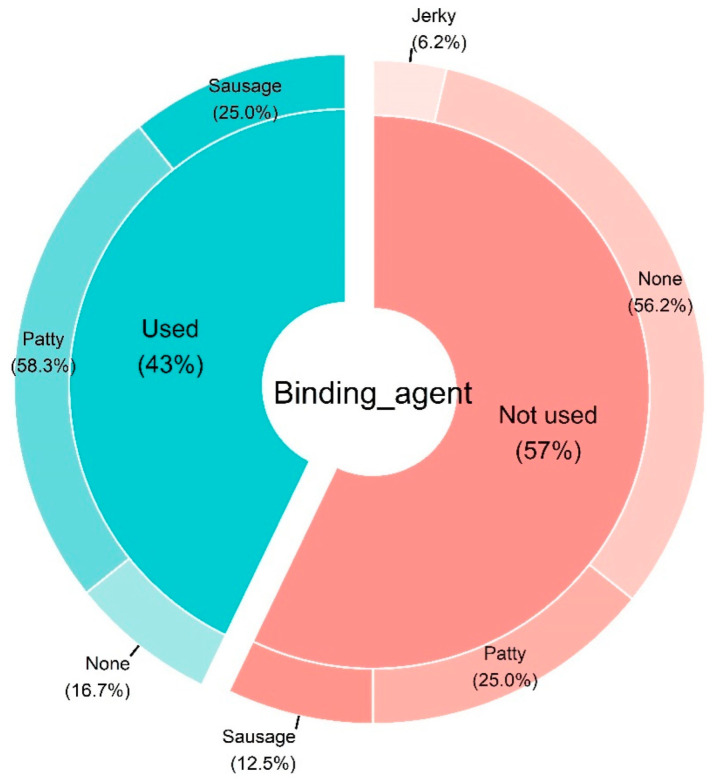
Pie donut chart summarizing the type of final product according to the use of binding agent from the studies included in the systematic review. The chart was constructed based on *n* = 28 articles.

Finally, patties (58.3%) and sausages (frankfurter) (25%) were more or less considered as the final product when a binding agent was used in the formulation (Figure 6).

**Figure 7:**
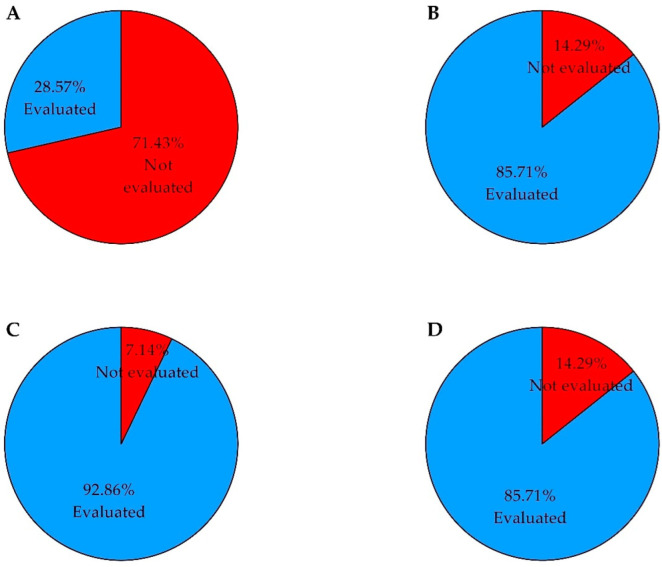
Pie charts showing the percentage of studies evaluating taste attributes (**A**), texture (**B**), sensory properties (**C**), physicochemical properties (**D**) in the systematic review. The chart was constructed based on *n* = 28 articles.

One of the main criteria for study inclusion was related to the evaluation of at least one quality characteristic. Indeed, Figure 7 presents the overall proportions of studies assessing the different categories of parameters.

Taste attributes were the least parameters reported by authors (28.57%) in all of the included studies. Textural profiles were evaluated in nearly about (85.71%), whereas sensory and physicochemical properties were reported (92.86%) and (85.7%), respectively, in all included studies.

Physiochemical characteristics were the second most evaluated set of parameters with a total of 83.3% of studies including them in their results. Finally, sensory and taste attributes were the least reported by authors with 46.7% and 43.3% of studies assessing them.

### 3.3. Quality Assessment

Quality assessment of current articles was performed based on standard established guidelines by Kmet, et al. [59]. The results of the quality assessment are presented in Table 2. The objective, study design sample size, analytic methods, results reported, and control confounding of the outcome assessor was reported in all the studies. The interventional random allocation, interventional and blinding investigators, and interventional blinding subjects’ outcome misclassification bias were not applicable in the included articles since these biases are related to randomized control trials in clinical studies. Moreover, the sample size appropriateness was partially described in the majority of studies. However, methods, control confounding, results, and conclusions were described in detail in most of the studies.

## 4. Discussion

TVP is a dry bulky commodity and is notably extracted from leguminous crops, such as soybeans, pea, and lupine which are produced by the cooking extrusion process. During the production of TVP, the protein is subjected to thermal and mechanical stresses by heating and applying high pressure to produce fibrous TVP [57]. The TVP production has been categorized into types based on water addition during the extrusion process. Low moisture extrusion (dry; <35% of water) and high-moisture (wet; >50% of water) types [57]. Sufficient literature is available on the production of TVP with high and low moisture extrusion [46,60,61]. Similarly, due to extensive agriculture practices, human nutrition has evolved from time to time consequently based on recent trends in agricultural production TVP has obtained popularity due to its outstanding quality characteristics concerning red meat. The most popular TVPs are produced from soy, pea, wheat, and rice protein. TVP can be used to replace meat or can be served in combination with meat at a certain level [62]. The USDA approves up to 30% of TVP in school lunch as mixed with ground beef patties. The TVP showed a resemblance with meat chewiness and flavor [9,63]. TVP has been used as a meat replacer with many economic and functional benefits [64]. Current literature indicates promising outcomes for partial or complete replacement of meat with TVP [19,20]. Previously, Samard and Ryu [45] and Bakhsh et al. [20] evaluated different types of meat, such as beef, pork, and chicken as compared to TVP-based meat analogs. The results indicate that TVP-based meat analogs were nearer to chicken and pork meats, respectively. Moreover, the different proteins had different quality characteristics, such as soy [65,66], and oat or pea dry fractioned textured isolate proteins which have been shown different implications [6,67,68].

It appears the majority of studies included in this systematic review were conducted in the Asian region, particularly Korea and China. Alternatives to meat as a protein source have existed for millennia, with traditional products, such as tofu and tempeh (made from soybeans) and seitan (made from wheat protein) used as affordable, functional, and nutritious protein sources as early as 965CE and originating in China [25,69].

The consumption of TVP in meat analogs in different regions of the world is based on cultural and religious reasons [7]. Vegetarian dishes including alternative proteins were frequently consumed in the Buddhist religion [70]. In 1960 the invention of TVP led to the modernization of meat analogs as TVP was used as a prime ingredient in the vegan version of meat alternatives [11,71]. The consumption of meat analogs and TVP production is relatively recent in western countries. However, due to personal preference, religious beliefs, and health awareness, the vegetarian and flexitarian population increased tremendously in recent years, particularly in western countries. Although, the consumer approval and demand for these meat alternatives are considerably low. Nevertheless, the production new generation of meat analog-like beyond burgers and impossible burgers has cleared the way to reach the table of western families [11,21]. This could be the reason that Asian countries, such as China and Korea, and Indonesia had more interest in academic research in meat analogs as compared to western countries. Likewise, the research related to meat analogs has surged in 2021 as compared to previous years.

As depicted in Figure 4, soy-based TVP was the most commonly used in studies of the current review. The demand for meat alternatives is growing in contrast to animal-derived proteins. Soy texturized protein, such as TVP, and TSP (texturized soy protein) has been used commonly in meat analogs due to tremendous emulsifying, fat absorption, and gelling qualities [23]. Soy products have better yields, easier handling, lower transportation, and preservation charges as compared to meat. A TVP-based diet is economically feasible and the high protein ingredients provide a variety of choices [72]. Furthermore, the appearance, flavors, and health impact of different proteins are different. Therefore, it is important to select a quality protein for the production of TVP and formulation of meat analogs. Nowadays several types of plant-based proteins are available for the manufacture of meat analogs. However, soy and pea protein are considered the best options due to the possession of some characteristics that resemble meat. The advantage of soy and protein-protein isolate, apart from the high protein purity, is its light color and bland flavor compared to the other protein. Soy-based TVP comprises its richer profile it includes 35% to 40% high-quality protein with a well-balanced composition of amino acids, 15% to 20% fat, 30% carbohydrate, and 10% to 30% moisture also rich in fiber, iron (Fe), calcium (Ca), zinc (Zn), and B vitamins [73]. Additionally, Soy-based TVP_S_ consists of low saturated fat, a high concentration of essential amino acids, low calories, and is cholesterol-free [74].

This review highlighted that in their trials, researchers tend to prioritize texture, and physicochemical properties more than any other quality characteristics (Figure 7). Indeed, in the formulation of meat analogs with the inclusion of TVP, the food producers face the biggest hurdle in the development of adequate texture and taste [75]. In meat analog formulation, the inclusion of simple protein does not guarantee the quality of taste, texture, and appearance. The reason behind the inclusion of extruded products, such as TVP and TSP is to achieve the desired taste and especially fibrous texture and visible appearance. The substitution of texturized proteins in meat analogs occurs in two ways. The first one is through blending and mixing with texturized proteins including meat and the other way is the complete incorporation of meat by TVP to form a meat analog [72]. Generally, the inclusive properties of meat analogs including taste, texture, and appearance are not improved with meat extender alone, however, mixed with meat they expand the overall functional characteristics of meat analogs manifolds. Furthermore, the quality and texture of raw materials can be enhanced by adding chemicals and ingredients through a process of texturization [72,76]. Soy protein isolates and concentrates, wheat gluten, egg white, and other binding agents, such as gelling agents and starches, are supplied to enhance the water-holding capacity, texture, and emulsification characteristics. As the texture and flavor are considered as primary factors which affect the consumer decision in the selection of meat analogs [26].

In the current study, a majority of studies were incorporated with binding agents. Binding agents in meat analogs can be ingredients of animal or plant origin that serve both as water and fat binder. Such substances include wheat gluten, milk proteins, eggs white, carrageenan, methylcellulose, xanthan gum, and other ingredients. Depending on the quantities added, some ingredients can interact as both binders and as extenders. Elevated water holding capacity, and protein network formation can be observed as the main function in the ingredients that have a higher amount of protein. Nevertheless, ingredients that have less or zero protein content, such as flours and starches, act as fillers, despite their water and fat binding traits through the physical entrapment [23]. The concentration level of the binding agents impacts the characteristics of the ultimate product. Besides protein binders, polysaccharides, such as pectin, guar gum, carrageenan, and methylcellulose, are recommended for use in meat-analog products as binders and extenders [77]. For instance, the gelling and thickening functions of polysaccharides, improvement of rheological properties, and water binding capacity have created a promising ingredient that can be implemented in the meat analog industry [78].

## 5. Limitations and Challenges

The current study is associated with some limitations. Indeed, based on inclusion criteria our study is limited to physicochemical, textural, and sensory characteristics of meat substitutes, whereas the nutritional and human health aspects as well the consumer acceptance of TVP-based meat analogs products are excluded in this systematic review. Additionally, we did not conduct a meta-analysis in the current study due to the dissimilarities between study designs.

The main purpose and ultimate challenge of making meat analogs is the production of a sustainable product that recreates conventional meat in all of its physical sensations. Normally, the meat substitute acquired from the current TVP-based extruded products is different from muscle tissue because it is deficient in terms of the fibrous structure, tenderness, and meaty sensation as real meat. Consequently, the major challenge for meat analog production is to acquire the texture and taste of real meat, which may necessitate exceptional designs for meat alternative formulations and the optimization of processing conditions. Seeking more low-cost premium plant protein sources and combinations of selected food ingredients for the preparation of meat-like products is crucial.

## 6. Conclusions

In conclusion, this study summarized the available evidence concerning the incorporation of TVP in the formulation of meat analogs. The various types of TVPs were discussed in relation to physicochemical, Textural, and sensorial characteristics. However, further studies pooling effect size via a meta-analysis approach should preferably be conducted to fill the gap in the current knowledge.

## Figures and Tables

**Table 1:** Studies characteristics.

Articles	Type of TVP Used	Binding Agent	Meat Type	Product Type	Quality Characteristics Evaluated
Sunchaleev et al., 2001 [34]	Soy	None	Beef	Patty	Physicochemical, organoleptic
Kim et al., 2011 [35]	Mushroom, Soy	Yes	Beef	Patty	Texture, Physiochemical
Liu et al., 2005 [36]	Soy	None	Pork	None	Texture, extrusion
Katayama et al., 2008 [37]	Soy	None	Chicken	None	Sensory, Texture, Physiochemical
Liu et al., 2008 [38]	Soy	None	None	None	Extrusion, Protein solubility
Pereira et al., 2011 [39]	Soy	Yes	Pork	Sausage	Texture, Physiochemical, sensory
Schäfer et al., 2011 [40]	Soy, Pea	Yes	Veal+ Pork	Sausage	Texture, Sensory, Gel strength
Gao et al., 2015 [41]	Soy	Yes	Pork	Patty	Texture, Thermo-rheology, chemical
Hidayat et al., 2018 [42]	Soy	Yes	Beef	Sausage	Physiochemical, Sensory, Texture
Ghribi et al., 2018 [43]	Chickpea	None	Turkey	Sausage	Physicochemical, Sensory, texture
Setiadi et al., 2018 [44]	Soy	Yes	Duck	None	Physicochemical, Texture
Samard et al., 2019a [45]	Soy, Wheat	None	Beef, Pork, Chicken	None	Physicochemical, Texture
Samard et al., 2019b [46]	Soy, Mung bean, Pea, Wheat	None	None	None	Physicochemical, Texture
Murillo et al., 2019 [47]	Pea	None	None	None	Diffusivity, Thermodynamics
Webb et al., 2020 [48]	Chickpea, Pea	None	Chicken, Beef	None	Physicochemical, Texture
Wi et al. et al., 2020 [49]	Soy	Yes	None	None	Physiochemical, Sensory, Texture
Bakhsh et al., 2021a [18]	Soy	Yes	Beef	Patty	Physiochemical, Sensory, Texture
Bakhsh et al., 2021b [20]	Soy	Yes	Beef, Pork	Patty	Physiochemical, Sensory, Texture
Bakhsh et al., 2021c [19]	Soy	Yes	Beef	Patty	Physiochemical, Sensory, Texture
Ball et al., 2021 [50]	Soy, Oat	None	Beef	Patty	Physicochemical, storage
Kim et al., 2021a [51]	Soy, Pea Lentils, Faba bean	None	Beef	Patty	Physiochemical, Sensory, Texture
Kim et al., 2021b [52]	Pea, Soy, Lentils, Faba beans	None	None	None	Physicochemical, Texture
Saerens et al., 2021 [53]	Soy, Pumpkin seed	None	Beef, chicken, Pork	Patty	Extrusion, Climate change,
Sakai et al., 2021 [54]	Soy	Yes	None	Patty	Physicochemical, Texture
Samard et al., 2021 [55]	Soy, Wheat gluten	Yes	None	Patty	Physicochemical, Texture
Kim et al., 2022 [56]	Soy, Insect	None	None	Jerky	Physicochemical, Tenderness
Lee et al., 2022 [57]	Rice, Soy, Wheat	None	None	None	Physiochemical, Texture, Extrusion
Yuan et al., 2022 [58]	Soy	None	None	Sausage	Physiochemical, Sensory, Texture

**Table 2:** Studies quality assessment.

Articles	1	2	3	4	5	6	7	8	9	10	11	12	13	14
Sunchaleev et al.2001 [34]	No	Partial	Partial	partial	N/A	N/A	N/A	Partial	Partial	Partial	No	No	Partial	Yes
Kim et al., 2011 [35]	Partial	Yes	Partial	Yes	N/A	N/A	N/A	Yes	Partial	Yes	No	No	Yes	Yes
Liu et al., 2005 [36]	Partial	Yes	Partial	Yes	N/A	N/A	N/A	Partial	Partial	Partial	yes	yes	Yes	Yes
Katayama et al., 2008 [37]	Yes	Yes	Yes	Yes	N/A	N/A	N/A	Yes	Partial	Yes	yes	Yes	Yes	Yes
Liu et al., 2008 [38]	Yes	Yes	Yes	Yes	N/A	N/A	N/A	Yes	Partial	Yes	No	No	Partial	Partial
Pereira et al., 2011 [39]	Yes	Yes	Partial	No	N/A	N/A	N/A	Partial	Partial	Yes	yes	yes	Yes	Yes
Schäfer et al., 2011 [40]	Partial	Yes	Yes	Yes	N/A	N/A	N/A	No	Partial	Partial	Partial	yes	Yes	Yes
Gao et al., 2015 [41]	Yes	Yes	Partial	Yes	N/A	N/A	N/A	Partial	Partial	Partial	No	yes	Yes	Yes
Hidayat et al., 2018 [42]	Yes	Yes	Partial	partial	N/A	N/A	N/A	No	Yes	Partial	No	yes	Yes	Yes
Ghribi et al., 2018 [43]	Yes	Yes	Yes	Yes	N/A	N/A	N/A	No	Yes	Yes	Partial	yes	Partial	Yes
Setiadi et al., 2018 [44]	No	Partial	Partial	No	N/A	N/A	N/A	No	Partial	No	No	yes	Partial	Yes
Samard et al., 2019a [45]	No	Yes	Yes	partial	N/A	N/A	N/A	No	Yes	Yes	yes	yes	Yes	Yes
Samard et al., 2019b [46]	Yes	Yes	Yes	Yes	N/A	N/A	N/A	Partial	Partial	Yes	yes	No	Yes	Yes
Murillo et al., 2019 [47]	No	Yes	Partial	Yes	N/A	N/A	N/A	Yes	Partial	Yes	yes	No	Yes	Yes
Webb et al., 2020 [48]	Yes	Yes	Yes	Yes	N/A	N/A	N/A	Partial	Partial	Yes	No	No	No	Yes
Wi et al. et al., 2020 [49]	Yes	yes	Yes	Partial	N/A	N/A	N/A	Partial	Yes	Yes	No	No	Yes	Yes
Bakhsh et al., 2021a [18]	Yes	Yes	Yes	Yes	N/A	N/A	N/A	Partial	Yes	Partial	No	yes	Yes	Yes
Bakhsh et al., 2021b [20]	Yes	Partial	No	Yes	N/A	N/A	N/A	Partial	Yes	Partial	yes	No	Yes	Yes
Bakhsh et al., 2021c [19]	Yes	Yes	No	Yes	N/A	N/A	N/A	Partial	Yes	Partial	yes	yes	Yes	yes
Ball et al., 2021 [50]	Yes	Yes	No	Yes	N/A	N/A	N/A	Partial	No	Partial	yes	yes	yes	Yes
Kim et al., 2021a [51]	Yes	Yes	Partial	Yes	N/A	N/A	N/A	Partial	Partial	Yes	yes	yes	Yes	No
Kim et al., 2021b [52]	Yes	Yes	Yes	Yes	N/A	N/A	N/A	Partial	No	Partial	No	yes	Partial	Yes
Saerens et al., 2021 [53]	Yes	Yes	Yes	Yes	N/A	N/A	N/A	Partial	Partial	Partial	yes	No	Yes	Yes
Sakai et al., 2021 [54]	Yes	Partial	Partial	Yes	N/A	N/A	N/A	Partial	Partial	Yes	yes	yes	Partial	Yes
Samard et al., 2021 [55]	Yes	Yes	Partial	Yes	N/A	N/A	N/A	Yes	No	Yes	No	yes	Yes	Yes
Kim et al., 2022 [56]	Yes	Yes	Partial	partial	N/A	N/A	N/A	Partial	No	Yes	No	No	Yes	Yes
Lee et al., 2022 [57]	Partial	Yes	Partial	Yes	N/A	N/A	N/A	Yes	No	Yes	No	yes	Yes	Yes
Yuan et al., 2022 [58]	Partial	Yes	Yes	Yes	N/A	N/A	N/A	Partial	Yes	No	No	No	Yes	Yes

Abbreviations: 1: Question/objective sufficiently described?, 2: Study design evident and appropriate?, 3: Method of subject/comparison group selection or source of information/input variables described and appropriate?, 4: Subject (and comparison group, if applicable) characteristics sufficiently described?, 5: If interventional and random allocation was possible, was it described?, 6: If interventional and blinding of investigators was possible, was it reported?, 7: If interventional and blinding of subjects was possible, was it reported?, 8: Outcome and (if applicable) exposure measure(s) well defined and robust to measurement/misclassification bias?, 9: Sample size appropriate?, 10: Analytic methods described/justified and appropriate?, 11: Some estimate of variance is reported for the main results?, 12: Controlled for confounding?, 13: Results reported in sufficient detail?, 14: Conclusions supported by the results?

## Data Availability

Not applicable.
